# Medical News and Miscellany

**Published:** 1893-10

**Authors:** 


### Medical News and Miscellany.

---

**Dr. J. D. Westervelt, Jr.**, has been appointed surgeon to the Mexican National Raitroad Co., at Alice, Texas.

---

**Pay day** has come: the JOURNAL "expects every man to do his duty." Do unto others, as you know how it is yourself, or words to that effect.

---

**Bennett** being the potentiality (that's the word now) of the Austin District Medical Society, there was no regular quarterly meeting held in September, he being absent at Polyclinic, New York.

---

**The Texas Medical Journal** has removed its office to the suite of rooms over Corner Bros. book store, upper Congress Avenue, where we will be pleased to receive our friends when they visit the city of the violet crown.

---

**Dr. Justus Duffau.**—The JOURNAL is pained to announce that Dr. Duffau, formerly a resident of this city, but for several years past a leading practitioner in Houston, died at his mother's home in Austin, on the 8th of October inst.

---

**Southern Surgical and Gynecological Association** will hold its next session in New Orleans on the 14th, 15th and 16th of November, prox. The medical profession are cordially invited to attend. Prospects are good for a splendid meeting.

---

**Fortnightly M. D.**—The Happy Medium offers a prize for every error found in its pages, and actually awarded a prize to a subscriber who pointed out that "substitution" *ought* to have been spelled "substitusion." Good joke on Webster.

---

**It Pays.**—The Secretary of the Louisville Medical College writes the JOURNAL, "Our class is larger than ever before. We have 500 matriculants, amongst them a great many Texans. *Thanks to your* JOURNAL. Write us earlier next year about space."

**Cinderella.**—Medical Director John L. Gihon, in his admirable address on "Motes and Beams," said that Hygiea is the Cinderella of the Medical family; like her she had been left at home neglected, and like her she was now receiving the homage of princes. He was glad he had lived to see her "souzeraine."

---

**The Pharmacentical** Department of the University of Texas was organized and begun work October 2. Faculty—James Kennedy, M. D., Ph. G., Professor of Pharmacy; S. M. Morris, M. D., B. Sc., Professor of Chemistry; Ed. Randall, M. D., Professor Materia Medica; James Kennedy, M. D., Ph. G., Lecturer on Botany.

---

**Dr. Ralph Steiner,** of Austin, Texas, has been appointed Consul to Munich by Mr. Cleveland. Dr. Steiner is worthy and deserving of the honor. He goes to Munich to study for two years, and applied for this position as a side line; the salary will pay all his expenses during his student life there; a good thing. The doctor was born with a silver spoon in his mouth.

---

**Sharp & Dohme,** the well-known manufacturing chemists so long established in Baltimore have removed their general offices to New York, No. 41 John street. There has been no change either in the personnel of the firm or the general business policy. Their branch office in Chicago is at 221 Randolph street. This firm is too well known to need any commendation at our hands. Its characteristic honesty and integrity have earned for the firm the name of "Old Reliable." Their goods are standard the world over.

---

**Transactions Texas State Medical Association:** April meeting, 1893, Galveston. The last volume of Transactions is out. It is bound uniformly with the series, in brown cloth and gilt, and contains 446 pages. It is a little larger than its immediate predecessor of '91–2. Dr. West, the Secretary, has done himself and the Association credit in the completeness and very neat appearance of this volume, and it ought to be highly prized by members. It contains a larger number of papers, and the general average of excellence is higher, we believe, than of late years. Among them are some really valuable contributions. Thirty-seven new members were added to the roll at the Galveston meeting, and some important changes were made in the by-laws.

---

**The New Orleans Polyclinic.**—It is very gratifying to note the continued prosperity of this home institution. It has demonstrated its claim to Southern patronage, has successfully met and overcome numerous obstacles thrown in its way, and now, with increased facilities for clinical teaching, and a wealth of material at the Great Charity, the Eye, and other hospitals, and a strong faculty, it makes its annual announcement to Texas physicians, through this, the Texan's pride. See advertisement.

Dr. Isadore Dyer, long connected with the Charity Hospital, and lecturer on Dermatology in the Tulane University Medical College, and editor of Department of Dermatology in TEXAS MEDICAL JOURNAL, has been elected Professor of Dermatology in the New Orleans Polyclinic.

---

**Surgical Instruments.**—A recent number of Meyer Bros. & Co.'s St. Louis *Drug Magazine* states that the Fort Worth Pharmacy Company, of Fort Worth, carries the largest stock of surgical instruments and mechanical curative devices in the Southwest. We are personally acquainted with these people, and have a correct knowledge of their stock, fully endorse the above, and will further say, their prices we know to be as low as any of the Eastern houses, and that they are now giving good satisfaction to five hundred Texas doctors whom they number as their patrons. A year ago we asked the profession to aid us in building up this house—a home institution—where orders could be promptly filled and delivered. We have now to say that satisfactory results are being obtained. The Fort Worth Pharmacy Company are also agents for three manufactories of electric Batteries and two manufactories of Physicians' Chairs. Write to them for what you may want.

---

**Mr. Ernest Hart,** while the guest of the American Medical Profession, was guilty of many things that, from an American standpoint of professional ethics, were unbecoming one so distinguished. Dr. W. A. Hammond, in a letter to the *Now York Medical Journal*, gave him an excoriating that it did one's soul good to read, and which must have made Mr. Hart feel that he had made an unmitigated ass of himself. Dr. Hammond had a good subject for his caustic pen, and he handled it, if not exactly in a manner to the Queen's taste, very much to our liking. We see he is now in a controversy with one Dr. Archie Stockwell. The "correspondence" between them (*N. Y. Med. Journal*) is *piquant* in the extreme,—on Dr. Hammond's side at least,—and his last is *so* caustic that we opine that of Mr. Archie there will not be left a greasy spot withal, when he comes out of the fight. We would as soon encounter a buz-saw as to get Hammond after us.

---

**Our Esteemed Contemporary,** Dr. H. H. Haralson, of the *Mighty Mississippi Medical Journal*, has just recovered from a long and severe spell of typhoid fever. The TEXAS MEDICAL JOURNAL extends its congratulations to the doctor and to the readers of his sprightly little "monthly."

By the bye, the July number of said s. l. m. contains an "original article" (*not* an editorial)—a paper, on "Modern Antipyretics," written by Dr. Haralson, and read for him by Dr. Geo. A. Herndon, before the Smith County, Mississippi, Medical Society, which paper does the author credit. It was well received and highly complimented. Notwithstanding the subject is very hackneyed, Dr. Haralson said a good many new and original things about it; he had had a large experience with the several agents mentioned, and had intelligently observed and interpreted the effects produced. Like all who have preceded him, Dr. Haralson is impressed with the danger which attends the injudicious use of these agents, especially antipyrine, and cautions his readers to be guarded in its administration, especially in repeated doses.

In dysmenorrhœa Dr. Haralson found antipyrine singularly useful. He says:

"I have had some experience in this line (with dysmenorrhœa), and am prepared to state that I have had good results from the use of antipyrine in this trouble, not palliative alone, but permanently good ones. In severe cases of dysmenorrhœa we are compelled to use remedies to alleviate the sufferings of our patients. The pain is sometimes so intolerable that patients are driven to the use of opiates, and I am free to say that I do not fear individual administration of these agents in this trouble so much as I do morphine.

"Dr. Eliza Mosher has used acetanilide in this trouble with good results. She claims to have used it in several hundred cases without any bad results. To avoid self-administration she has adopted the plan of having the powders ready and giving them herself without a prescription. By adopting this plan the patient is not only not able to prescribe them for herself, but she cannot prescribe them for her friends. My experience is not so extended with antipyrine, but the few cases I have treated with this drug have in the main been benefited. I usually prescribe three or four grains to the dose, to be begun about two days before the expected period, and given every four hours during the day, until the period has passed. I, of course, do not rely upon this drug to correct certain conditions that may be productive of this trouble, but rely upon it more especially to tide the patient over these periods, which are so torturing to them. It is safer than morphine, and better; better, because there is so much less danger of self-administration, while, by commencing early, as good results are obtained from its use. Besides this, there is a class of these cases permanently benefited by this drug. I refer to cases of spasmodic dysmenorrhœa. I have been treating two cases recently, and using very little else besides antipyrine, and my patients, both, are greatly improved. Sometimes it is very difficult to ascertain the cause, and until this is done, there is no drug so free from danger that promises so much.

"I cannot give my endorsement to the administration, at one dose, of twenty grains of antipyrine. I do not believe that it is ever necessary to give it in such large doses, besides, observation has demonstrated that it is dangerous to use the drug so heroically."

---

### THE DOCTOR'S LAMENT.*

TO HIS LADY-LOVE.

Your life leads down by peaceful, tranquil rivers,
Whose shady bank the cool sea breeze invites;
While mine, alas, is spent midst torpid livers,—
And similar sad and melancholy sights.

To you, the perfumed air is rich with sounds
As sweet as when first Sappho's harp was strung;
While I, in sun and dust must take my weary rounds
To feel a pulse, or view a coated tongue.

The choicest books beguile your leisure hours
And soothe to sleep or 'wake to sympathetic tears;
But woe is me,—I spend my feeble powers
'Midst fever's fervid heat, or checking diarrhœas.

You sleep in peace on soft and downy beds,
And dream, perhaps, of flowers in sunlit lands;
While I, no doubt, am soothing aching heads,
Or humbly giving aid by pulling hands.

Your lovers kneel before you in rapturous adoration,
And tales of love in mellifluous measures pour;
Creditors besiege me; they are my abomination,
And moneyless patients daily throng my office door.

---

*This appeared in one of our very early numbers, in 1885, and is here reproduced by request, revised and slightly changed.—F. E. D.

Thy gentle pen, anon, the choicest thoughts indict,
   That dwell within thy gentle breast, or tender memory fosters;
Prescriptions I, with stubby pencil write,
   "Recipe; misce et fiat haustus."

Riches I bring thee not, to pride's exactions fill,
   Nor offer thee, as I could wish, a handsome marriage portion;
Wilt thou despise my only store—a pill?
   Or deign to take, perchance, a pharmaceutical lotion?

Alas, alas, my lady-love, I tire indeed of these
   Old scaly scalps of seborrhœa and eczematous hands;
Let's trim our sails to catch an outward breeze,
   And endosmose in pleasant foreign lands,—

Away beyond the seas, on some peaceful starlit isle,
   Where rythmic wavelets break on coral strands;
There'll be no more of fever, pus nor bile,—
   As down the happy years we'll pull each other's hands.

F. E. DANIEL, M. D., Austin, Texas.

---

### THE COUNTRY DOCTOR.

Prof. Wm. Keiller, in his address to the matriculants of the Texas Medical College, in Galveston, on the opening day of the present session, drew the following beautiful picture of the country doctor.

Prof. Keiller said: "Now in closing I wish to present to you a picture of the goal for which you are striving, a little glimpse of one of the noblest lives that men lead on earth.

"In the winter of 1890 there appeared in the Royal Academy exhibition, in London, a picture which attracted large crowds of warm admirers, and stirred many of them to their heart's core. Luke Fildes had been asked by a patron to paint a picture illustrative of English life, and he chose "The Doctor" as his subject, and painted to the life the ideal representative of the most humane of professions. No prosperous, wealthy specialist is this hero of the medical world. The first glance at the man reveals the general practitioner, the hard-worked country doctor. The only daughter of a humble farmer, a wee, curly-haired lassie of two or three, is sick, well nigh unto death. It is no long, wasting disorder—she is too plump for that; but the crisis of an acute malady. The next few hours will probably decide her fate. It is evening, and the doctor has seen her in the morning, and now, tired by a long round of visits, he has called again; and his energies, fatigued as he drove along in his buggy, are again called into full activity by the exigencies of the case before him. Poor wee pet! She is very low. Her pulse is rapid and feeble, her respiration quick and shallow; but, if her heart can be kept up a little longer, she may get a little sleep and ultimately pull through. Our doctor has with his own hands given her her medicine, and now sits watching its effect. Already she is soothed, her pulse is a little slower and firmer, her respiration less hurried, and she has dropped into a brief doze.

"And while she sleeps let us look at the doctor. He is the central figure of the painting, the center of interest even to the stricken father and mother, because in his hands is the life of their first-born. A man of strong and massive build, about 40, and therefore in the zenith of his mental vigor, with fifteen to twenty years' experience to mature his judgment; no dude, but well dressed, as self-respect demands of all men of well balanced minds; the whole man reveals the well-to-do country practitioner. And his face? Genial, thoughtful, and above all things reliable and strong. There is no weakness there.

"It is late, and he is tired, but there is no languor in his attitude; he has forgotten all the world,—wife, children, the cosy supper waiting for him an hour or more, the father and mother of the child, all are forgotten but the patient and his science. He is not absorbed over an interesting case which will be worth reporting at the local society; he is face to face with a problem of life and death. No debasing thought of a big fee (I am almost ashamed in the face of such a scene to mention the word)—I say, no thought of his fee mars the current of his calculations. No big fee can come out of that small cottage; but a little laughing face that was the sweetest sunbeam of the young couple's hearth may shine again or set to-night, and the doctor's skill may decide the matter.

"In the background the young mother, wearied with watching, has yielded her place to the man in whom, next to God, her trust is placed; and has laid her head upon the table, half in despair, half in unuttered prayer. Her husband stands beside her, his hand, with a subtle sympathy that she only can fully understand, on his wife's shoulder; a touch that means hope for the best, sympathy and strength for the worst, his face expressive of the calmer way in which strong men meet life's worst vicissitudes.

"Gentlemen, it is well that in commencing a new course of life each man should have an ideal, and should keep his ideal ever before his mind. And I would hold up before you as a high ideal this picture of the country practitioner. Few of you will ever have the opportunity to become great specialists; but all of you may become most excellent men and good physicians, and lead lives which shall call down on your heads the blessings of a grateful people. The man whom our artist has chosen is a student, every inch of him; a true man of science, but his science is a means to relieve suffering humanity. He is no money grabber; no dispenser of drugs at so much a bottle; no smooth, honey-tongued ladies' doctor with half hours to spare listening in scented, half lit rooms and cushioned lounges to a languid account of imaginary ailments. He is no brilliant specialist, who has, by hard, up-hill work and much self-denial it may be, or by some lucky stroke of fortune, reached that pinnacle of success where life is easy, leisure frequent, successes brilliant, and fees often princely. He his strong; strong because of his knowledge, because of the hard, honest work of his student days, because though he may not have carried off honors he has determined to understand everything he has studied and make it his own; strong because since his college days he has remained always a zealous student; strong because it is his nature to be so. Such a physician must be reliable. His word must be slowly given, but when given as good as fulfilled; his word must be his bond; his tongue and his whole life must speak the truth. Our ideal physician must be benevolent—generous even to a fault, but just also. No warm-hearted waster of his goods and energies on all and sundry, but ready ever to err on the side of generosity and great-heartedness where there shall appear a fitting object for his compassion.

"He must be unselfish. No life grates more on the selfish than the physician's. He, more constantly than any other man, is called on to sacrifice self, health, wife and children, home comforts and home duties. It is not on the specialist that all this falls most heavily; it is on the general practitioner.

"In short, gentlemen, the life before you is one of labor from the beginning. If you wish to make fortunes do not study medicine. Patients you may have in plenty, and your pockets all the time be very empty, aye, even gratitude may be often denied you. But you have chosen a profession, than which none is more full of scientific interest, no study is more fascinating, no life gives more constant opportunity for the highest moral culture. And now and then you will get a handshake from a husband or father, a look from a wife or mother, that will make you feel that life is worth living after all, and you had rather be a doctor than anything else on earth."

